# Anti-Inflammatory Activity of *Populus deltoides* Leaf Extract via Modulating NF-κB and p38/JNK Pathways

**DOI:** 10.3390/ijms19123746

**Published:** 2018-11-25

**Authors:** Ye Eun Jeong, Mi-Young Lee

**Affiliations:** 1 Department of Medical Science, College of Medical Science, Soonchunhyang University, Asan, Chungnam 31538, Korea; jye0564@naver.com; 2 Department of Medical Biotechnology, College of Medical Science, Soonchunhyang University, Asan, Chungnam 31538, Korea

**Keywords:** anti-inflammation, *Populus deltoides* leaf, iNOS, MAPK, NF-κB

## Abstract

*Populus deltoides*, known as eastern cottonwood, has been commonly used as a medicinal plant. The aim of the present study was to investigate the mechanism underlying the anti-inflammatory activity of *P. deltoides* leaf extract (PLE). PLE effectively inhibited the expression of inducible nitric oxide synthase (iNOS) and nitric oxide (NO) production in lipopolysaccharide (LPS)-stimulated RAW 264.7 cells, but not that of cyclooxygenase-2 (COX-2) and prostaglandin E2. Proinflammatory tumor necrosis factor alpha (TNF-α) levels were also reduced by the extract. PLE inhibited the phosphorylation of nuclear factor-kappa B (NF-κB) and inhibitor of Kappa Bα (IκBα), and blunted LPS-triggered enhanced nuclear translocation of NF-κB p65. In mitogen-activated protein kinase (MAPK) signaling, PLE effectively decreased the phosphorylation of p38 and c-Jun N-terminal protein kinase (JNK), but not of extracellular signal-regulated kinase 1/2 (ERK1/2). Taken together, these results suggest that anti-inflammatory activity of *P. deltoides* leaf extract might be driven by iNOS and NO inhibition mediated by modulation of the NF-κB and p38/JNK signaling pathways.

## 1. Introduction

Inflammation is a complex defense mechanism for an organism against a range of harmful stimuli, including bacteria and parasites. Macrophages are important immune cells responsible for innate cellular immunity involved in host defense and immunity against foreign stimuli [1]. Macrophages stimulated by lipopolysaccharide (LPS), a principal component of the outer membrane of Gram-negative bacteria, regulate inflammation by releasing proinflammatory cytokines, such as tumor necrosis factor alpha (TNF-α), interleukin-6 (IL-6), and adhesion enzymes, such as inducible nitric oxide synthase (iNOS) and cyclooxygenase-2 (COX-2) [1,2,3]. In addition, the mitogen-activated protein kinase (MAPK) and the nuclear factor-kappa B (NF-κB) signaling pathways are important intracellular molecular pathways leading to inflammatory responses to LPS stimulation in macrophages [4,5]. However, imbalanced production of proinflammatory cytokines by macrophages causes destructive inflammation in the body and leads to diseases, such as cancer [6], atherosclerosis [7], diabetic nephropathy [8,9], and neurodegenerative disorders [10].

Nitric oxide (NO) induced by iNOS has been known to be a mediator and regulator of inflammatory reactions that can provoke harmful effects to host tissues. Therefore, inhibiting the production of NO, TNF-α, IL-6, and IL-8 secreted by macrophages through blocking the expression of iNOS can serve as the basis of anti-inflammatory drug development [11,12].

MAPK rapidly responds to a variety of extracellular stimuli and regulates essential cellular events to control a vast array of physiological processes, including macrophage-mediated inflammatory responses [13,14]. The MAPK signaling pathway, involving extracellular signal regulated kinase 1/2 (ERK1/2), c-Jun N-terminal protein kinase (JNK), and p38, is activated by sequential phosphorylation events, and mediates signaling cascades leading to the activation of various transcription factors, such as NF-κB [15,16]. Activation of the inhibitor of Kappa B kinase (IKK) complex, especially IKKβ, results in the breakdown of inhibitor of NF-κB (IκB) following inflammatory stimuli through direct phosphorylation of inhibitor of Kappa Bα (IκBα). NF-κB binds to the IκB family in the form of homo- or heterodimers, and the phosphorylated IκBs are subsequently ubiquitinated and degraded by the proteasome, leaving NF-κB free to translocate to the nucleus. Nuclear NF-κB binds to cognate enhancer/promoter elements of inflammation-related target genes [17,18,19]. Various anti-inflammatory agents targeting the MAPK and NF-κB pathways are being developed on the basis of this cascade response.

A variety of natural products with anti-inflammatory activity have been studied worldwide, including those derived from medicinal plants, such as *Allium hookeri* [20], Moutan cortex [21], *Salvia officinalis* [22], *Perilla frutescens* [23], *Camellia japonica* [24], and *Nauclea officinalis* [25]. Moreover, the therapeutic potential of plant secondary metabolites is regarded as a promising research target in the pursuit of novel, natural anti-inflammatory drugs. Some plant-based natural drugs are in clinical use, and some are undergoing clinical trials [26]. A wide variety of chemical components in plants, such as phenolic compounds, flavonoids, coumarins, alkaloids, saponins, sterols, terpenoids, and essential oils, have been known to exhibit anti-inflammatory activities by inhibiting the molecular targets of pro-inflammatory mediators in inflammatory responses. Notably, phenolic glycosides were known to possess a wide variety of bioactivities, including antioxidant, antimicrobial, and anti-inflammatory activities [27,28,29,30].

*Populus deltoides*, also called eastern cottonwood, is distributed worldwide and has been utilized in traditional medicine to treat various inflammatory conditions [31]. The bark tincture of *P. deltoides* is known to be effective for treating rheumatism and gout, and its buds have been used to treat colds, respiratory problems, myalgia, and soreness [32]. In addition, various chemical components, including salicortin, salicin, salicyl alcohol, pyrocatechol, 1-*O*-*p*-coumaroyl-β-d-glucoside, populoside, ω-salicyloylsalicin, chrysin-7-glucoside, deltoidin, and tremulacin, were identified in *P. deltoides* [33,34,35,36]. However, information on the utility and efficacy of *P. deltoides* leaf as an anti-inflammatory agent is limited. In this study, we investigated the anti-inflammatory activity of *P. deltoides* leaf extract, and dissected the mechanism underlying its anti-inflammatory activity in LPS-induced RAW 264.7 macrophages.

## 2. Results

### 2.1. Effect of P. deltoides Leaf Extract on LPS-Stimulated iNOS and NO in RAW 264.7 Cells

**Figure 1:**
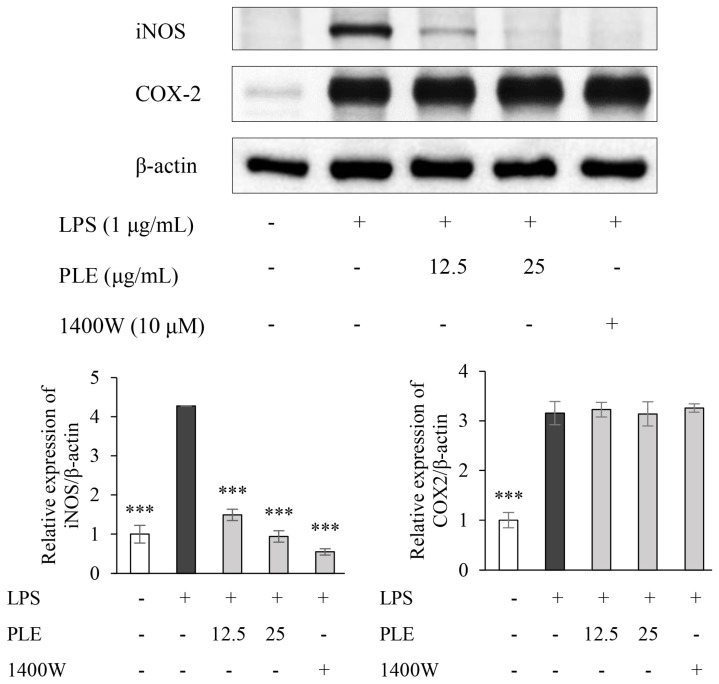
Effects of *P. deltoides* leaf extract on inducible nitric oxide synthase (iNOS) and cyclooxygenase-2 (COX-2) expression in LPS-stimulated RAW 264.7 cells. The cells were treated with *P. deltoides* leaf extract (12.5 and 25 μg/mL) in the presence of 1 μg/mL LPS for 24 h. RAW 264.7 cells were treated with 10 μM of iNOS inhibitor 1400 W simultaneously with LPS treatment. Error bars represent the mean ± SD. *** *p* < 0.001 compared to LPS alone.

Proinflammatory enzyme iNOS produced NO, which has a marked impact on various acute and chronic inflammatory diseases [37]. Figure 1 shows the effect of PLE on the expression of iNOS and COX-2. The LPS-induced expression of iNOS in RAW 264.7 cells was significantly reduced by application of 12.5 and 25 μg/mL of PLE. Conversely, COX-2 expression was not affected by PLE at these concentrations. The LPS-induced iNOS expression was also inhibited by the iNOS inhibitor 1400 W. 

**Figure 2:**
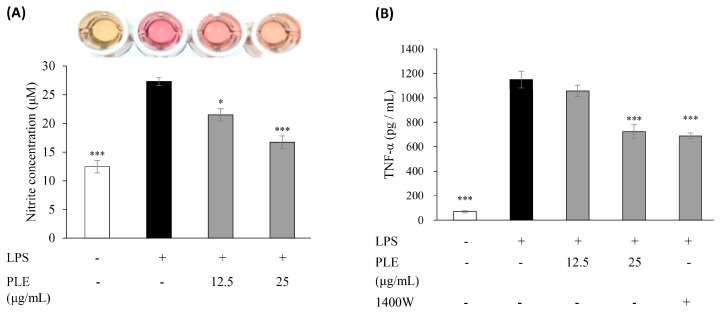
Effect of *P. deltoides* leaf extract on the production of nitric oxide (NO) and tumor necrosis factor alpha (TNF-α) in LPS-stimulated RAW 264.7 cells. *P. deltoides* leaf extract (12.5 or 25 μg/mL) suppressed (**A**) NO production and (**B**) TNF-α expression in RAW 264.7 cells stimulated by 1 μg/mL LPS. The expressions of TNF-α were also down regulated by treatment of iNOS inhibitor 1400 W (10 μM). Error bars represent the mean ± SD. * *p* < 0.05 and *** *p* < 0.001 compared to LPS alone.

Figure 2A shows that PLE significantly reduced LPS-induced NO production. The effect of PLE on the level of TNF-α, a pro-inflammatory cytokine, in LPS-stimulated RAW 264.7 cells was also investigated by ELISA. The level of LPS-induced TNF-α was markedly reduced after application of 12.5 and 25 μg/mL of PLE (Figure 2B). Moreover, iNOS inhibitor 1400W also notably reduced TNF-α expression. Taken together, the results suggest the anti-inflammatory activity of PLE might be exerted by reducing iNOS expression and NO production, not COX-2.

### 2.2. Effects of P. deltoides Leaf Extract on the NF-κB Signaling in LPS-Stimulated RAW 264.7 Cells

**Figure 3:**
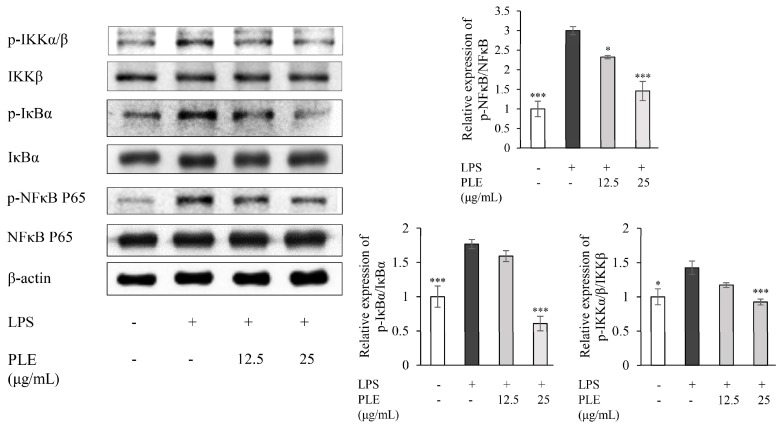
Effects of *P. deltoides* leaf extract on the LPS-induced activation of the nuclear factor-kappa (NF-κB) pathway in RAW 264.7 cells. The cells were treated with *P. deltoides* leaf extract (12.5 or 25 μg/mL) in the presence of 1 μg/mL LPS for 24 h. Error bars represent the mean ± SD. Values of * *p* < 0.05 and *** *p* < 0.001 compared to LPS alone were considered statistically significant.

In order to examine whether the NF-κB pathway is involved in the anti-inflammatory effect of PLE, the expression pattern of NF-κB signaling molecules was investigated by western blotting. Figure 3 shows that the phosphorylated IKKα/β, IκBα, and NF-κB p65 were enhanced in LPS-stimulated RAW 264.7 cells. However, the phosphorylation of IKKα/β, IκBα, and NF-κB p65 was significantly reduced upon treatment with PLE. This result suggests that PLE effectively suppresses the NF-κB-associated inflammatory response induced by LPS.

**Figure 4:**
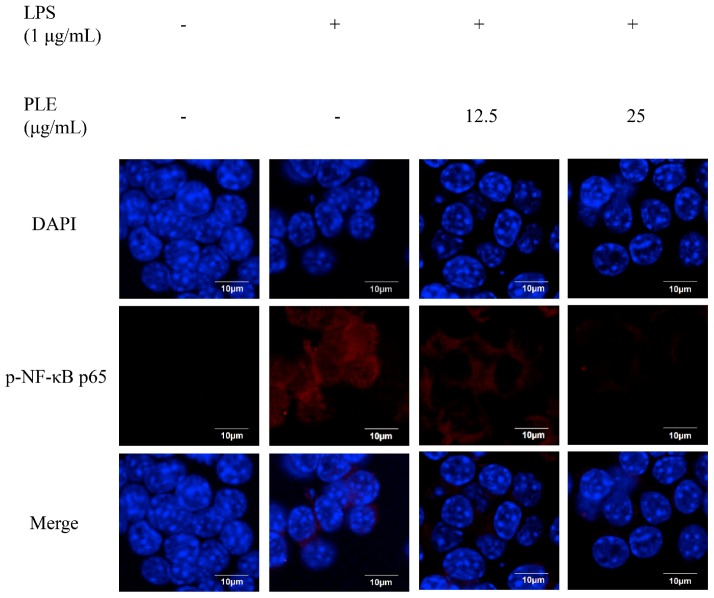
The inhibitory effect of *P. deltoides* leaf extract on NF-κB p65 translocation in LPS-stimulated RAW 264.7 cells. Representative photomicrographs of immune-labelled NF-κB (red) and nuclear counterstaining with DAPI (blue) in LPS-stimulated RAW 264.7 cells treated without and with 12.5 or 25 μg/mL of PLE. LPS increased colocalization of NF-κB and DAPI in RAW 264.7 cells. However, PLE reduced the nuclear translocation and accumulation of NF-κB, which was induced by LPS. Scale bar = 10 μm.

Generally, phosphorylated NF-κB p65 follows a nuclear translocation in LPS-stimulated RAW 264.7 cells [38]. As shown in Figure 4, LPS-triggered nuclear translocation and accumulation of p-NF-κB p65 were dramatically inhibited by PLE, compared to the LPS-stimulated group.

### 2.3. Effects of P. deltoides Leaf Extract on MAPK Signaling in LPS-Stimulated RAW 264.7 Cells

**Figure 5:**
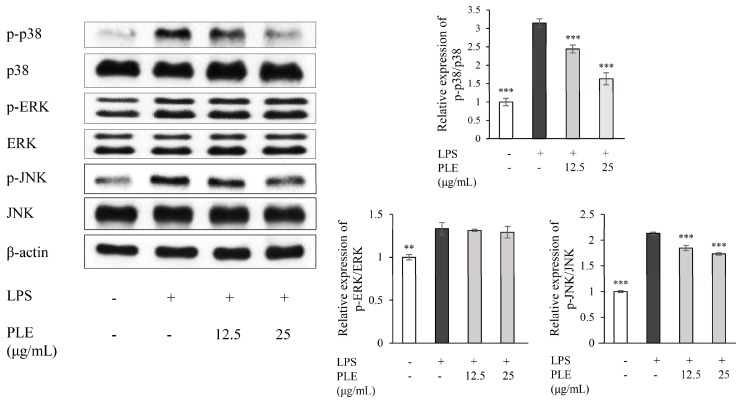
Effects of PLE on the phosphorylation of mitogen-activated protein kinase (MAPK) cascade (p-ERK1/2, p-JNK, and p-p38) in LPS-stimulated RAW 264.7 cells. The cells were treated with *P. deltoides* leaf extract (12.5 or 25 μg/mL) in the presence of 1 μg/mL LPS. Error bars represent the mean ± SD. Values of ** *p* < 0.01, and *** *p* < 0.001 compared to LPS alone.

The mitogen-activated protein kinase (MAPK) family, including ERK1/2, p38, and JNK, play an important role in the regulation of gene expression, cellular growth, and survival through association with the inflammatory response. Figure 5 indicates that the phosphorylation of JNK and p38 was upregulated by LPS, but the phosphorylation of JNK and p38 was markedly down regulated by application of 12.5 and 25 μg/mL of PLE. Interestingly, however, phosphorylation of ERK1/2 was not influenced by PLE application. 

## 3. Discussion

*P. deltoides* has been widely used as an ancient medicinal herb. However, recent studies on *P. deltoides* have mainly focused on its response to environmental stressors, including air pollution [39] and water deficits [31], supporting the virtues of *P. deltoides* as a roadside tree. In this investigation, the mechanism underlying the anti-inflammatory effect of PLE was examined in RAW 264.7 cells stimulated with LPS. 

In response to diverse stimulants including microbial infection, immune cells actively release NO, which may lead to chronic or acute inflammatory diseases [40,41]. Our results show that PLE reduced LPS-induced iNOS expression and NO production to a level similar to that of the control. The overproduction of NO is positively linked with excess iNOS expression. Thus, the release of NO was reduced as iNOS expression was blocked [42]. Accordingly, selective inhibitors of iNOS in inflammatory macrophages are considered to be effective therapeutics for inflammatory diseases [43]. In this investigation, PLE was very effective in inhibiting iNOS expression and NO production, showing its superior anti-inflammatory activity. In addition to NO, prostaglandin E2 (PGE_2)_ released from immune cells plays a major role in a variety of inflammatory responses catalyzed by the expression of COX-2 [44]. NO produced by iNOS in the inflammatory response contributes to upregulation of COX-2 via crosstalk between NO, COX-2, and PGE2 [45]. However, PLE did not inhibit the expression COX-2 or prostaglandin E2 in the present study. 

The genus *Populus*, including *Populus deltoides*, is characterized by the presence of salicin, salicortin [33,46,47], pyrocatechol [48], 1-*p*-coumaroyl-β-d-glucoside [49], and tremulacin [50]. Salicin is a commonly found phenolic glycoside in the bark, leaves, and buds of more than 100 *Populus* species [35] with anti-inflammatory, antiangiogenic, and antitumor activities [51,52]. Salicin has been widely used in natural medicines without side effects, such as allergic skin reactions and vomiting [53]. However, salicin itself does not possess anti-inflammatory properties until it is metabolized into salicylic acid in the blood and gastrointestinal tract. Interestingly, some derivatives of salicylic acids showed differential inhibitory effects on COX-2 in murine macrophages [54].

Although PLE might include various potential COX-2 inhibitors, the anti-inflammatory effect of PLE was exerted through iNOS and NO, not COX-2, in this study. This result could be explained by the property of plant extracts. The possession of many constituents might be determined by the presence of synergic interaction and cocktail effect of the extracts. Similar effects were found in the extracts of *Acanthopanax sessiliflorum*, *Daphne genkwa*, and *Thuja orientalis*, strongly inhibiting iNOS activity, but weakly inhibiting COX-2 activity [55]. Generally, plant extracts as a mixture of phytochemical components have been suspected to have higher activities than those obtained with single isolated components. In some cases, the activity of a mixture of pure compounds was not higher than that of a single compound [56]. Therefore, minor constituents of plant extracts may play a major role in expressing specific activity of the extracts.

TNF-α is crucial for inducing synergy in NO production in LPS-stimulated macrophages and induces inflammatory responses such as vasodilatation, edema, and fever [57,58]. In this study, PLE significantly inhibited the levels of TNF-α. The MAPK and NF-κB signaling pathways are known to be important in regulating the expression of proinflammatory enzymes and cytokines. NF-κB is a major transcription factor involved in the regulation of proinflammatory cytokines, such as p50, p65, and IκBα [59,60]. In this investigation, the inhibition of p65 and IκBα phosphorylation, along with nuclear translocation by PLE, demonstrated that the inflammatory response of PLE is associated with NF-κB signaling. 

The MAPK family of proteins, which consist of p38, JNK, and ERK1/2, positively affect NF-κB activation [61]. Interestingly, we confirmed by western blot that PLE significantly inhibited p38 and JNK phosphorylation dose-dependently, while ERK1/2 phosphorylation was not significantly affected. MAPK kinase (MAPKK), the upstream effector that activates MAPK, is composed of MEK 1-7 [62,63]. MEK1 and MEK2, the upstream regulators of ERK1/2, were not affected by PLE (data not shown). Similar anti-inflammatory properties were reported in sophocarpine from *Sophora alopecuroides*, angelicin from *Psoralea corylifolia* L. fruit, and the sulfated derivative of 20(*s*)-ginsenoside Rh2 from red ginseng, which inhibit p38 and JNK phosphorylation, but not ERK1/2 [12,59,62]. 

**Figure 6:**
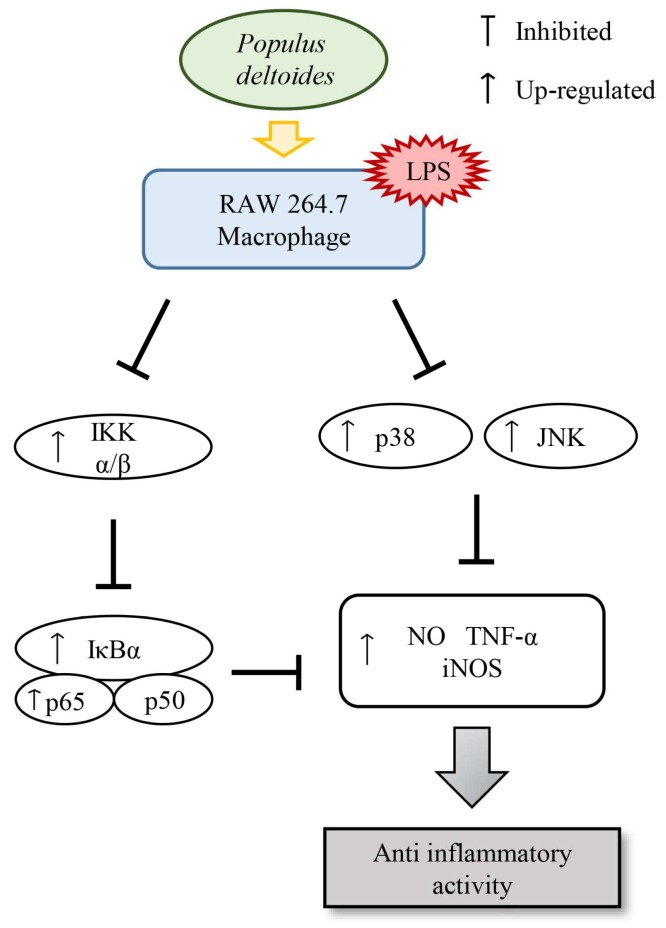
Schematic diagram of the suppressive effects of *P. deltoides* leaf extract on the inflammatory response via modulating NF-κB and p38/JNK pathway in LPS-stimulated RAW 264.7 cells.

Our data demonstrated that *P. deltoides* leaf extract exerts an anti-inflammatory effect, which might be attributed to the inhibition of iNOS expression, via suppression of the JNK and p38 MAPK signaling and the inhibition of NF-κB activation (Figure 6).

## 4. Materials and Methods

### 4.1. Preparation of Methanol Extract of P. deltoides

The leaf extract of *P. deltoides* was obtained from the Plant Extract Bank at the Korea Research Institute of Bioscience and Biotechnology (KRIBB, Daejeon, Republic of Korea) and was extracted with 99.9% pure methanol. The extract was dissolved in dimethyl sulfoxide to be 2% and stored at −20 °C until needed. The doses of the extract (12.5 or 25 μg/mL) used here were the highest concentrations that did not show any toxicities on the LPS-stimulated RAW 264.7 cells.

### 4.2. RAW 264.7 Cell Culture and Cell Viability Assay

RAW 264.7 cells, a murine macrophage cell line, were maintained in Dulbecco’s modified Eagle’s medium (DMEM; Hyclone, Logan, UT, USA) [with 10% fetal bovine serum (FBS; Hyclone, Logan, UT, USA) and 1% penicillin-streptomycin (PS; Hyclone, Logan, UT, USA)] in a humidified atmosphere containing 5% CO_2_ at 37 °C. Cells were stimulated by replacing the culture medium with medium containing 1 μg/mL LPS (Escherichia coli 011:B4, Sigma Chemical Co., St. Louis, MO, USA), and incubated with *P. deltoides* leaf extract for 24 h.

Cell viability was assessed by 3-(4,5-dimethyl-thiazol-2-yl)-2,5-diphenyltetrazolium bromide (MTT) assay. Briefly, 4 × 10^4^ RAW 264.7 cells per well of a 96-well plate were incubated with various concentrations of *P. deltoides* leaf extract (0–200 μg/mL) at 37 °C for 24 h and then the medium was completely removed. The cells were washed and treated with 50 μL of MTT, after which the plates were incubated at 37 °C in the dark for 3 h. The resultant formazan crystals were dissolved in 50 μL of dimethyl sulfoxide, and the absorbance was measured at 570 nm using an ELISA reader (Tecan, Männedorf, Switzerland).

### 4.3. NO Production 

The nitrite content in culture medium was measured as an indicator of nitric oxide (NO) production, according to the Griess reaction. Each supernatant (100 μL) was mixed with the same volume of Griess reagent (1% sulfanilamide in 5% phosphoric acid and 0.1% naphthylethylenediamine dihydrochloride in distilled water); the absorbance of the mixture at 570 nm was determined with an ELISA reader.

### 4.4. ELISA

The effect of PLE on TNF-α production in LPS-stimulated RAW 264.7 cells was measured by ELISA. TNF-α in the culture supernatant was quantified with an OptEIA mouse TNF-α (Mono/Mono) set (BD Biosciences, San Jose, CA, USA). The TNF-α level was calculated based on the standard curve, using the standard in the mouse TNF (Mono/Mono) set.

### 4.5. Western Blotting

Proteins were separated by 10% SDS–PAGE, then transferred onto polyvinylidene fluoride membrane (Bio-Rad, Hercules, CA, USA). After blocking for 2 h at room temperature with 5% bovine serum albumin (BSA), the membranes were incubated overnight at 4 °C with primary antibodies (iNOS, COX-2, IKKβ, p-IKKα/β, IκBα, p-IκBα, NF-κB p65, p-NF-κB p65, p38, p-p38, ERK1/2, p-ERK1/2, JNK, p-JNK (Cell Signaling Technology, Inc., MA, USA), and β-actin (Santa Cruz Biotechnology, Inc., Dallas, TX, USA)), which were diluted following the manufacturer’s recommendations. The membranes were then washed in Tris-buffered saline Tween 20 (TBST) and incubated with the appropriate HRP-conjugated secondary antibody (1:5000) at room temperature for 1 h. Protein bands were visualized using the Sensi-Q 2000 (Lugen Sci, Bucheon, Korea). The intensity of the bands was analyzed using ImageJ and normalized to β-actin.

### 4.6. Confocal Microscope Analysis

LPS-stimulated RAW 264.7 cells were treated with 12.5 or 25 μg/mL of *P. deltoides* leaf extract, after which the cells were fixed with 4% paraformaldehyde in phosphate-buffered saline (PBS) for 10 min. The cells were washed 3 times with PBS containing 0.5 mM MgCl_2_ and 1 mM CaCl_2_ for 5 min. After permeabilization with 0.1% Triton X-100 for 10 min, samples were incubated with blocking buffer (5% BSA in PBS). The cells were incubated with primary antibodies (p-NF-κB p65) (1:200) in 1% BSA for overnight at 4 °C. The cells were washed 3 times in PBS for 5 min and were stained for another 3 h with goat anti-rabbit IgG Texas red (1:1000) (Santa Cruz Biotechnology, Dallas, TX, USA). Cells were counterstained with 4, 6-diamidino-2-phenylindole dihydrochloride (DAPI) (Bio-Rad, Hercules, CA, USA) for 10 min to label the nuclei. The prepared cells were then observed under a confocal microscope (Olympus FV10i, Olympus, Tokyo, Japan), and images were recorded.

### 4.7. Statistical Analysis 

Data were entered into a Microsoft Excel datasheet, then transferred into the IBM Statistical Package for Social Sciences (SPSS, version 20, IBM SPSS statistics, Chicago, IL, USA). Data from each group are expressed as mean ± standard deviation (SD) of triplicate experiments. Group differences were analyzed using one-way analysis of variance (ANOVA), followed by a Bonferroni test and *p*-values less than 0.05 were considered statistically significant.
